# Occurrence of silk stitch abscess after surgery in patients with oral squamous cell carcinoma

**DOI:** 10.4317/medoral.18792

**Published:** 2013-05-31

**Authors:** Noriaki Yamamoto, Yoshihiro Yamashita, Daigo Yoshiga, Ayataka Ishikawa, Kou Matsuo, Ikuya Miyamoto, Masafumi Oda, Tatsurou Tanaka, Shinji Kito, Yuji Seta, Tetsu Takahashi, Hirofumi Koga, Kenji Kawano, Yasuhiro Morimoto

**Affiliations:** 1 DDS PhD, DDS PhD, Department of Dentistry and Oral-Maxillo-Facial Surgery, Oita University, Oita, Japan; 2 DDS PhD, Department of Oral and Maxillofacial Surgery, Fukuoka Dental College, Fukuoka, Japan; 3 DDS PhD, DDS PhD, DDS PhD, Division of Oral and Maxillofacial Surgery, Kyushu Dental University, Kitakyushu, Japan; 4 DDS PhD, DDS PhD, DDS PhD, Department of Health Improvement, Kyushu Dental University, Kitakyushu, Japan; 5 DDS PhD, DDS PhD, DDS PhD, DDS PhD, Division of Diagnostic Radiology Science, Kyushu Dental University, Kitakyushu, Japan; 6 MD PhD, Kitakyushu PET Center, Nishinippon Sangyoeiseikai, Kitakyushu, Japan; 7 DDS PhD, Center for Oral Biological Research, Kyushu Dental University, Kitakyushu, Japan

## Abstract

Objectives: To elucidate the predisposing factors and clinical characteristics related to the occurrence of stitch abscess after surgery in patients with oral squamous cell carcinoma (SCC). Patients and Methods: The subjects were 232 patients who underwent excision and/or reconstruction and/or neck dissection for oral SCC using silk sutures for high ligation of the blood vessels. Detection rates and characteristics of patients with stitch abscess were retrospectively evaluated by comparing patients with and without stitch abscesses after surgery diagnosed by ultrasonography and findings of various modalities in 232 patients. Several echogenic dots with subtle acoustic shadows in a hypoechoic mass were identified as the characteristic findings of stitch abscess on US. The patient groups with and without stitch abscess were compared with respect to various factors to identify those that predispose to the occurrence of stitch abscess. The factors analyzed included patients’ sex and age, chemotherapy treatment, radiotherapy treatment, the presence of a history of allergy, and blood test results. Results: A significant correlation was found between the occurrence of stitch abscess and age, liver function abnormalities on blood tests, and the presence of a history of allergy. Multiple stitch abscesses clearly tended to occur more often than single ones in patients with stitch abscess. Conclusions: The occurrence of stitch abscesses was related to age, liver dysfunction, and/or the presence of allergies. When diagnosing stitch abscess, the occurrence of multiple stitch abscesses is important.

** Key words:**Stitch abscess, oral cancer, predisposition, characteristics, squamous cell carcinoma.

## Introduction

A stitch abscess, which is an abscess that forms due to infection of sutures, is a noteworthy complication after various kinds of surgical procedures (1-7). Using non-absorbable silk sutures increases the risk of infection because they react with the connective tissue, causing adhesions around the stitch (5). Following surgery for malignant tumors, it has been very difficult to differentiate among stitch abscess, metastatic lymph nodes, and recurrence of malignancy (6). Therefore, it is important to be able to identify the factors that predispose to and the characteristics of stitch abscesses. However, to the best of our knowledge, there have been no previous reports examining the predisposition to and providing a clinical analysis of stitch abscesses, except for our imag-ing-related report and case reports (3,5-11). In the present study, various data, including sex, age, and blood test results, were retrospectively analyzed after surgery in patients with oral SCC to identify the clinical characteristics and other factors that predispose to the occurrence of stitch abscesses.

## Patient and Methods

The subjects were 232 patients (149 males, 83 females) who underwent excision and/or reconstruction and/or neck dissection for SCC of the oral cavity from 2004 to 2011 at Kyushu Dental College Hospital. In all cases where the original operative information was available, 2-0 or 3-0 silk was used for high ligation of the blood vessels.

**Figure 1:**
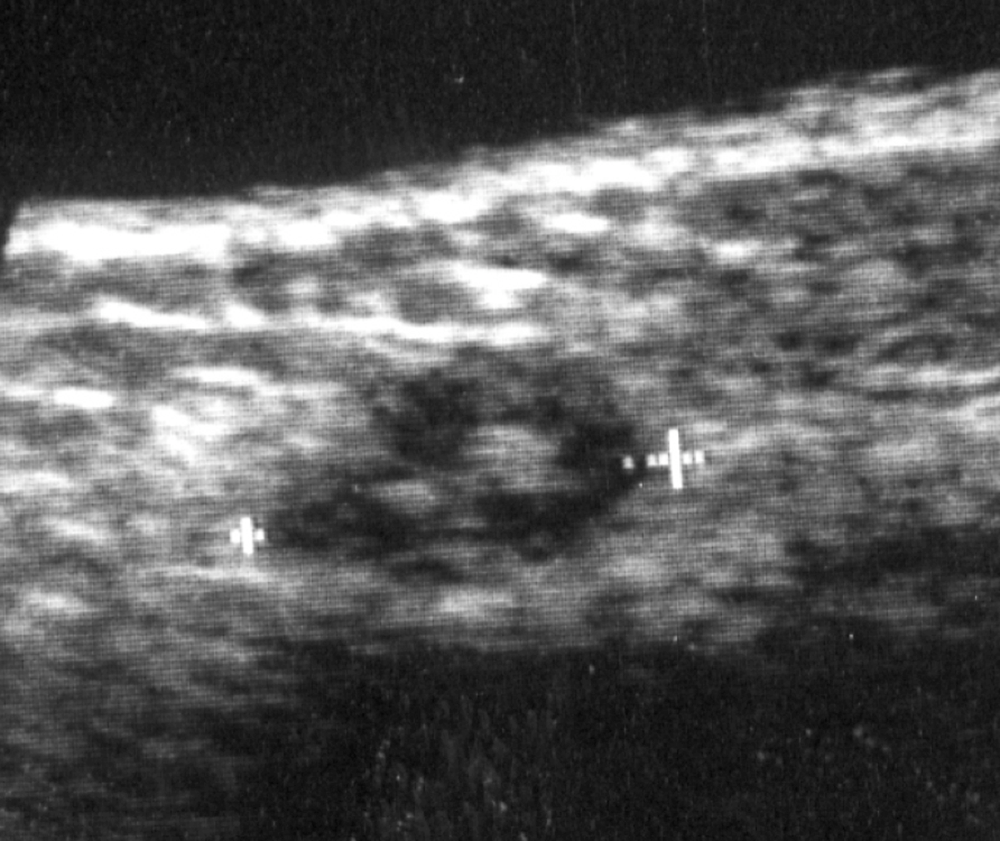
Typical US finding of a stitch abscess in the left submandibular space of a 38-year-old man 5 months after surgery for left tongue carcinoma. The image demonstrates several echogenic dots with subtle acoustic shadows in a hypoechoic mass (arrow).

All 232 patients were retrospectively divided into two groups based on the presence or absence of stitch abscess on US. To retrospectively diagnose stitch abscesses in the 232 patients with oral SCC, several echogenic dots with subtle acoustic shadows in a hypoechoic mass were identified as the characteristic findings of stitch abscess on US, as identified by Yamamoto et al. (Fig. 1) (6). The changes in stitch abscesses on subsequent US examinations were analyzed retrospectively. Of course, findings on various imaging modalities such as computed tomography (CT), magnetic resonance imaging (MRI), and positron emission tomography (PET)-CT using fluorine-18-labeled (18F) fluoro-2-deoxy-D-glucose (FDG) were also used. However, cases with masses and swelling that disappeared within 1 month and masses and swelling diagnosed as non-tumor recurrence and/or non-metastatic lymph nodes were excluded as non-stitch abscess.

The patient groups with and without stitch abscess were compared with respect to various factors to identify those that predispose to the occurrence of stitch abscess. The factors analyzed included patients’ sex and age, chemotherapy treatment, radiotherapy treatment, the presence of allergy, and blood test results.

All statistical analyses, such as Student’s t-test and the Chi-square test, were performed using SPSS™ software, version 11 (SPSS Inc., Chicago, IL, USA). Results were considered significant at p<0.05. In this study, the Human Investigations Committee of Kyushu Dental College protected individuals’ rights.

## Results

The incidence and imaging characteristics of stitch abscesses after surgery in patients with oral SCC.

The patients’ characteristics are shown in  Table 1. The overall 5-year survival rate was 81.1%. In addition, the occurrence rate of metastatic lymph nodes and the recurrence rate of primary tumors within 1 year were 6.5% and 6.9%, respectively. Overall, 20 (8.6%) of the 232 patients were diagnosed as having stitch abscesses based on the presence of specific findings of stitch abscess, such as several echogenic dots with subtle acoustic shadows in a hypoechoic mass (Fig. 1).

**Table 1:**
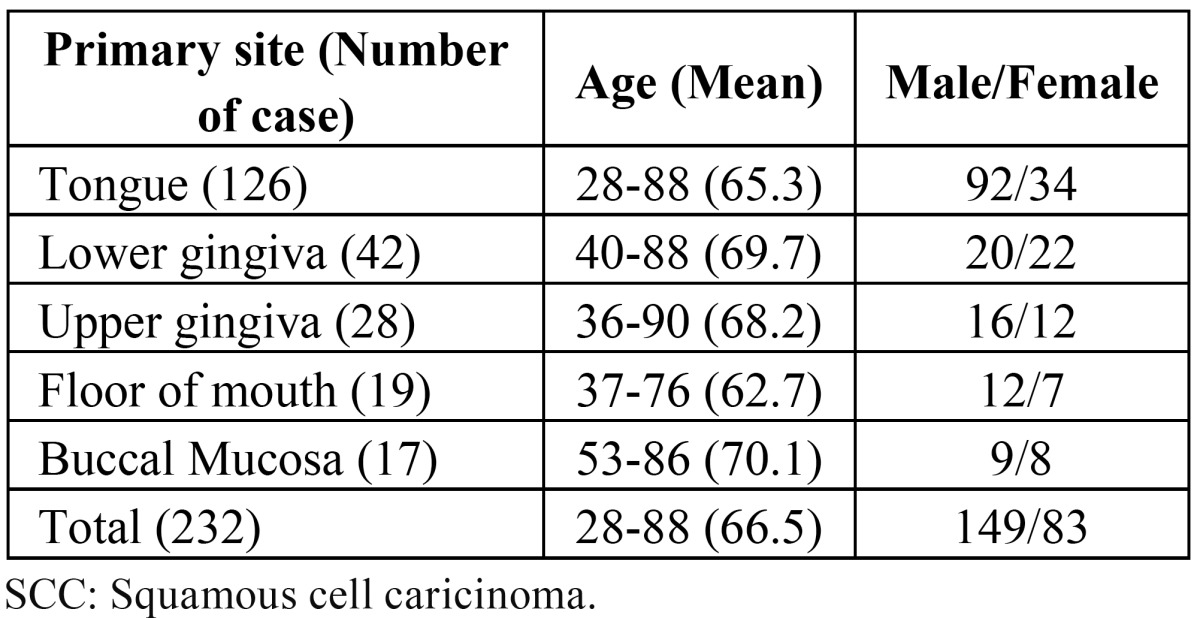
Anatomical distribution and features of oral SCC. patients.

Factors predisposing to and characteristics of stitch abscesses after surgery in patients with oral SCC.

The relationships between various factors and the occurrence of stitch abscesses after surgical procedures in patients with oral SCC are shown in  Table 2. A significant correlation was found between the occurrence of stitch abscess and age (Student’s t-test; p=0.0009), a history of allergy (?2 test; p=0.026), or liver dysfunction (?2 test; p=0.005). Patients with a stitch abscess tended to be significantly younger. An ALT over 30 IU/L and/or an AST over 40 IU/L were taken as indicating liver dysfunction.

**Table 2:**
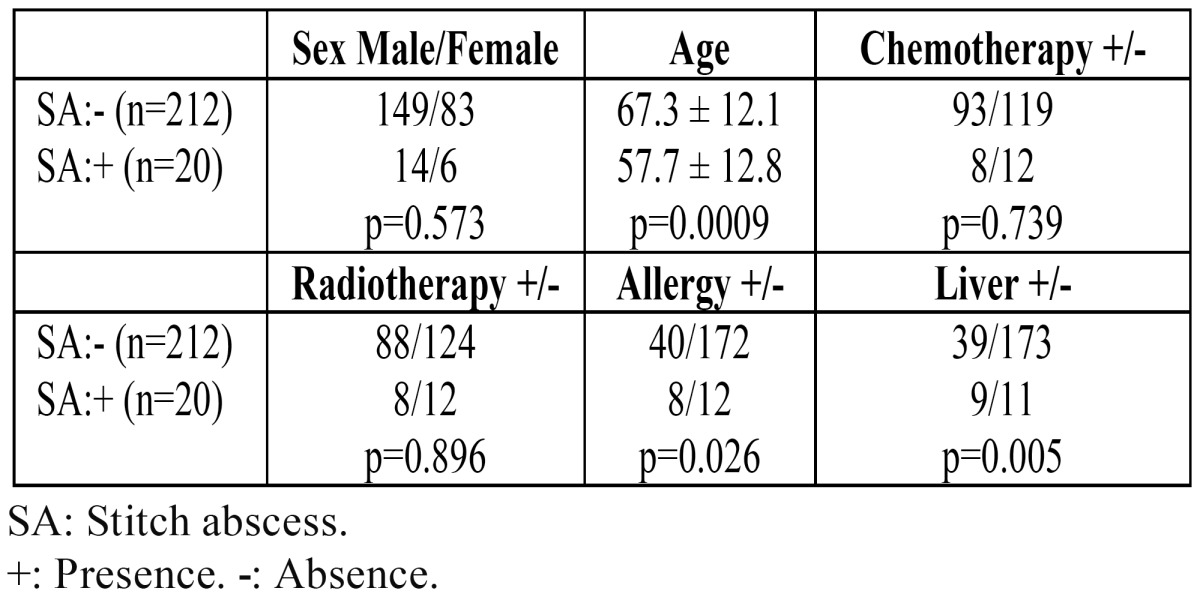
The relationships between various factors and the occurrence of stitch abscess.

However, there were no significant differences in sex (?2 test; p=0.573), the presence or absence of chemotherapy for oral cancer (?2 test; p=0.739), and the presence or absence of radiotherapy for oral cancer (?2 test; p=0.896).

Detection and follow-up of stitch abscesses on subsequent US examinations.

**Figure 2:**
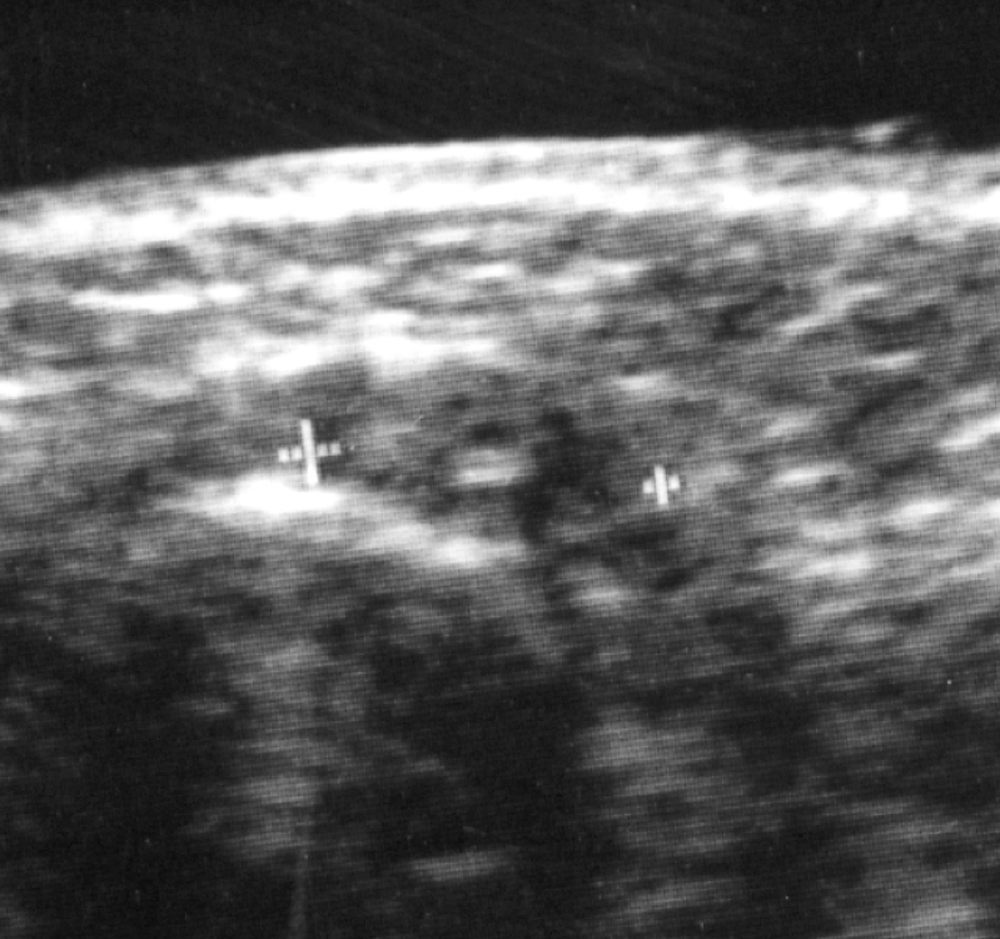
Resolution of a stitch abscess during US follow-up of a 58-year-old man with right tongue carcinoma. Typical stitch abscess (arrow) in the right submandibular space on US is apparent 6 months after surgery for right tongue carcinoma.

**Figure 3:**
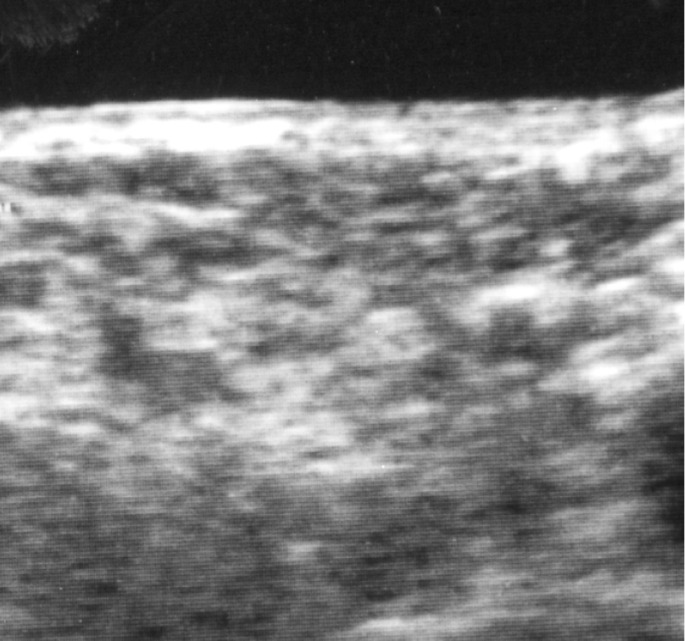
Resolution of a stitch abscess during US follow-up of a 58-year-old man with right tongue carcinoma. The mass has disappeared 12 months after surgery for right tongue carcinoma during follow-up (arrow).

The 20 patients with stitch abscesses developed them from 2 months to 1 year after surgery ( Table 3). One developed within 3 months, 7 from 3 to 6 months, and 12 from 6 months to 1 year. In 19 of the 20 patients, multiple stitch abscesses were detected. In 4 patients, surgical removal of the stitch abscess was performed, after which recurrence of the stitch abscess was not detectable on US. In 4 patients without surgical procedures, the mass disappeared spontaneously with disintegration of the stitch abscess in 4 patients, and the silk sutures appeared. After that, no recurrence of stitch abscess was detectable on US. In 2 patients, the masses disappeared spontaneously for unknown reasons within 1 year after the surgical procedures (Figs. 2,3). In 15 patients, the masses were detectable without changes in size, shape, and characteristic echoic findings over two years after surgery.

**Table 3:**
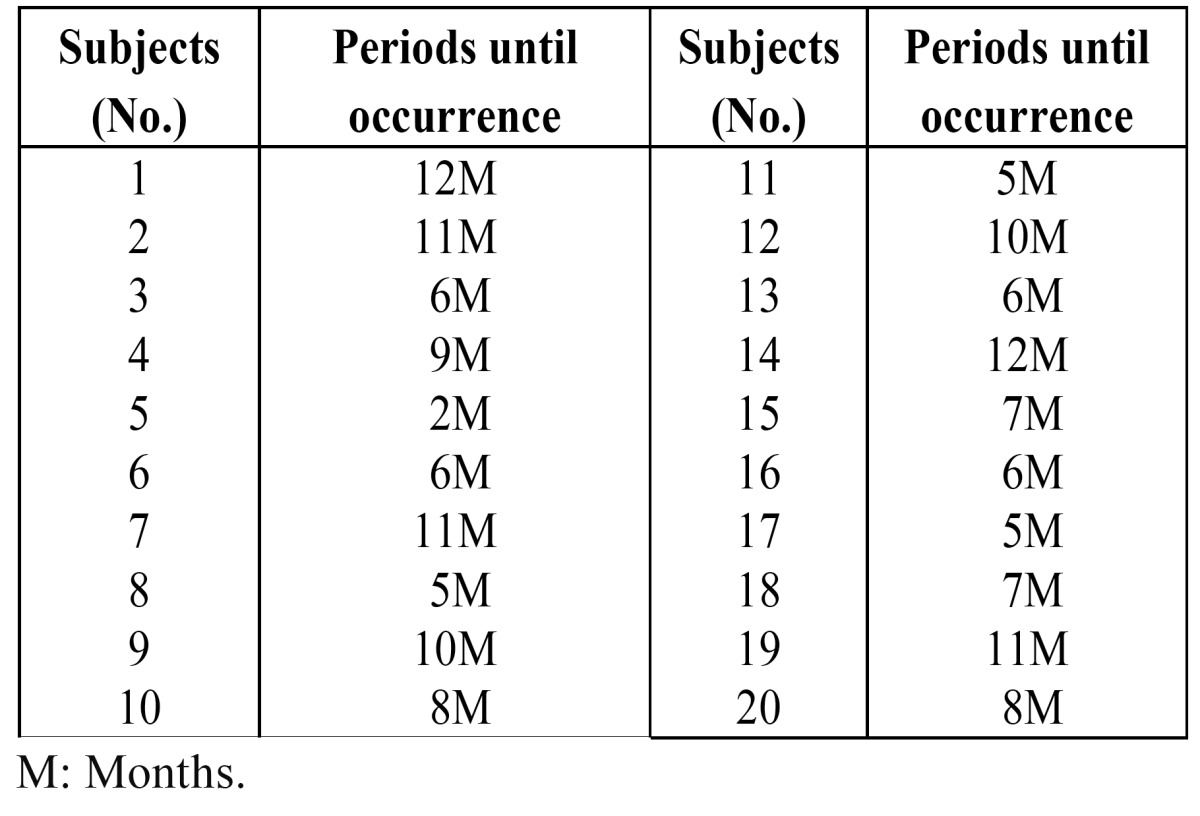
Distribution of the time to occurrence of stitch abscess.

## Discussion

The most important results of the present study are that significant correlations were found between the occurrence of stitch abscess and age, liver dysfunction, and a history of allergy by retrospective comparison between patient groups with and without stitch abscess. These results indicate that age, liver dysfunction, and a history of allergy are predisposing factors to stitch abscesses. A stitch abscess is not only an infectious response, but it is pathologically produced by the combined effects of the im-munologic response to sutures and the infectious response (12). In the allergic response, younger persons are more reactive to allergens than older persons (13). Therefore, we hypothesized that younger patients may mount a stronger allergic response to sutures as alien substances. As expected, there was a significant correlation between the presence of a history of allergy and the occurrence of stitch abscesses in the present study. In addition, liver dysfunction was also related to the occurrence of stitch abscesses. This implies that a poorer general condition decreases detoxification and lowers metabolism (14). The production of stitch abscesses might be related to not only the allergic response, but also to unknown alterations that occur with liver dysfunction. If so, the pathogenesis of stitch abscess may be mainly the result of the immunologic response to the suture, not the combined effect of the immunologic response and the infectious response. Silk sutures should not be used in surgical procedures involving patients who are relatively younger, have a history of allergy, and have liver dysfunction in order to avoid stitch abscesses.

Another important result of the present study was the finding that spontaneous alteration of stitch abscesses was demonstrated. Some stitch abscesses resolved spontaneously within 1 year after surgery. Four of them had disintegrated by themselves. There has been no previous report of such clinical findings, making this the first such report. In addition, those that had remained had a long axis of about 1 cm and were not growing worse. We cannot adequately explain how spontaneous resolution of a stitch ab-scess occurred. When local immuno-reactive effects gradually weaken with time, the stitch abscess might resolve. Further study is needed to resolve this issue. The present results certainly suggest that the clinical approach for stitch abscess does not require surgical treatment, but follow-up should be the primary approach.

An important additional finding was that multiple stitch abscesses are significantly more common than single stitch abscesses. Therefore, if one stitch abscess is found on US, a search for other stitch abscesses is needed. At the same time, the tendency for multiple entities is a very important finding in the diagnosis of stitch abscess. As in our previous report, all stitch abscesses oc-curred from 2 months to 1 year after surgery (6). Some cases of stitch abscess occurred following indirect hernia repair using silk suture over a 3-year period (3,4). Of course, further follow-up of these patients is needed, but we should pay attention to the early occurrence of stitch abscesses following surgery for oral SCC.

In the previous reports, including ours, the incidence rate of stitch abscess ranged from 0.6% to 12% (3,6,7). In the present study, the incidence rate of stitch abscess was 8.6%, relatively similar to the rate in previous reports following other surgical procedures (3,6,7). If non-absorbable silk sutures are used in surgical procedures for oral SCC, it is inevitable that stitch abscesses will occur in about 8% of patients as a complication. At present, since other types of suture material are likely to be equally efficacious in closing blood vessels, oral and maxillofacial surgeons should consider using non-braided or absorbable suture material for high ligation of blood vessels to prevent complications such as stitch abscesses. Therefore, surgeons should try to avoid using silk su-ture in various procedures, including surgery for patients with oral SCC, as soon as possible. At the same time, information about the complications of non-absorbable silk sutures needs to be widely disseminated through reports on the occurrence of stitch abscess after various surgeries.

The present study had several limitations. First, the sample size was relatively small, and only 20 patients developed stitch abscesses. Therefore, only a limited analysis was possible. Another limitation is that bacteriological data could not be obtained from all patients with stitch abscesses.

## References

[1] Adams IW, Bell MS, Driver RM, Fry WG (1977). A comparative trial of polyglycolic acid and silk as suture materials for accidental wounds. Lancet.

[2] Kronborg O (1976). Polyglycolic acid (Dexon) versus silk for fascial closure of abdominal incisions. Acta Chir Scand.

[3] Nagar H (1993). Stitch granulomas following inguinal herniotomy: a 10-year review. J Pediatr Surg.

[4] Calkins CM, St Peter SD, Balcom A, Murphy PJ (2007). Late abscess formation following indirect hernia repair utilizing silk suture. Pediatr Surg Int.

[5] Togo S, Kubota T, Takahashi T, Yoshida K, Matsuo K, Morioka D (2008). Usefulness of absorbable sutures in preventing surgical site infection in hepatectomy. J Gastrointest Surg.

[6] Yamamoto N, Yamashita Y, Tanaka T, Ishikawa A, Kito S, Wakasugi-Sato N (2011). Oral Oncol.

[7] Redaelli C, Niederhauser U, Carrel T, Meier U, Trents O (1992). Rupture of the Achilles tendon--fibrin gluing or suture?. Chirurg.

[8] ImamoÄlu M, Cay A, Sarihan H, AhmetoÄlu A (2005). Paravesical suture granuloma simulating a local recurrence of the immature sacrococcygeal teratoma. J Pediatr Surg.

[9] Imamoglu M, Cay A, Sarihan H, Ahmetoglu A, Ozdemir O (2004). Paravesical abscess as an unusual late complication of inguinal hernia repair in children. J Urol.

[10] Khan MW, Aziz MM (2010). Experience in laparoscopic cholecystectomy. Mymensingh Med J.

[11] Hsu TC, Wang CL, Wang TG, Shieh FJ (1998). Sonographic detection of a stitch abscess. J Clin Ultrasound.

[12] Javed F, Al-Askar M, Almas K, Romanos GE, Al-Hezaimi K (2012). Tissue reactions to various suture materials used in oral surgical interventions. ISRN Dent.

[13] Gupta R, Sheikh A, Strachan DP, Anderson HR (2004). Burden of allergic disease in the UK: secondary analyses of national databases. Clin Exp Allergy.

[14] Bosilkovska M, Walder B, Besson M, Daali Y, Desmeules J (2012). Analgesics in patients with hepatic impairment: pharmacology and clinical implications. Drugs.

